# Molecular and Microscopic Detection of *Enterocytozoon bieneusi* Among People Living With HIV in Northwest Iran: A Cross‐Sectional Study

**DOI:** 10.1155/japr/5245628

**Published:** 2026-11-04

**Authors:** Fares Bahrami, Mohammadbagher Khademerfan, Erfan Sherifi, Yahya Maroufi, Kambiz Karimi, Amir Abdoli

**Affiliations:** ^1^ Zoonoses Research Center Research Institute for Health Development Kurdistan University of Medical Sciences Sanandaj Iran muk.ac.ir; ^2^ Cellular and Molecular Research Center Research Institute for Health Development Kurdistan University of Medical Sciences Sanandaj Iran muk.ac.ir; ^3^ Department of Parasitology and Mycology Faculty of Medicine Kurdistan University of Medical Sciences Sanandaj Iran muk.ac.ir; ^4^ Department of Parasitology Faculty of Medical Sciences Tarbiat Modares University Tehran Iran modares.ac.ir

**Keywords:** *Enterocytozoon bieneusi*, Iran, microsporidia, molecular detection, One Health, people living with HIV, zoonotic potential

## Abstract

Microsporidian infections are increasingly recognized in humans and animals, with *Enterocytozoon bieneusi* having a broad host range and potential for zoonotic transmission. Data on its occurrence and molecular characteristics among immunocompromised populations in Northwest Iran remain limited. We investigated microsporidian infection among 161 people living with HIV (PLWH) in Kurdistan Province, Northwest Iran, using microscopic examination and nested PCR targeting the small subunit ribosomal RNA (SSU rRNA) gene. Microsporidia were detected in 10/161 participants (6.2%) by PCR and 8/161 (4.9%) by microscopy. All PCR‐positive specimens were identified as *E*. *bieneusi*, while no *Encephalitozoon* spp. were detected. In a paired analysis of the same specimens, PCR and microscopy showed 97.5% agreement (Cohen^′^s kappa = 0.765), and the difference in positivity rates was not statistically significant (McNemar′s exact test, *p* = 0.625). One PCR‐positive amplicon was successfully sequenced and showed 98%–100% nucleotide identity with sequences reported from human, animal, and environmental sources. Phylogenetic analysis placed this sequence among sequences from different host and environmental sources, indicating genetic similarity but not establishing a zoonotic transmission pathway. No significant associations were observed between infection and the demographic variables examined. The detection of *E*. *bieneusi* in this population, together with its genetic similarity to sequences from animal and environmental sources, supports further investigation of potential shared transmission sources. However, the molecular characterization remains preliminary because only a single SSU rRNA sequence was obtained. Human, animal, and environmental sampling combined with more discriminatory genetic markers would help clarify the epidemiology and transmission of *E*. *bieneusi* in Northwest Iran.

## 1. Introduction

*Enterocytozoon bieneusi* is an obligate intracellular microsporidian that infects humans and a broad range of animal hosts. In humans, infection is predominantly intestinal, although extraintestinal involvement has been described, particularly in severely immunocompromised individuals [1, 2]. Among people living with HIV (PLWH), microsporidiosis has been associated with gastrointestinal disease and may occur in the setting of advanced immunosuppression. *Encephalitozoon* species also infect humans and have a broader tissue distribution, with the potential for extraintestinal and disseminated infection [2–4].

*E. bieneusi* has been detected in domestic animals [5, 6], livestock [7], wildlife [8], birds [9], companion animals [5, 6], and environmental soil [10] and water sources [11], raising the possibility of transmission across human, animal, and environmental interfaces [12, 13]. The parasite is transmitted predominantly through the fecal–oral route, with contaminated water and food recognized as important vehicles of infection [1, 2]. Molecular studies have identified genetically related *E. bieneusi* sequences in humans and animals, supporting investigation of potential zoonotic and environmental transmission pathways [2–4].

In Iran, the reported prevalence of microsporidian infection among PLWH has ranged from 6% to 23% [14–18]. Molecular epidemiological data from Kurdistan Province remain limited despite extensive agricultural and livestock activity in the region and frequent human–animal contact. A recent study from the same region detected *E. bieneusi* in five of 116 patients with multiple sclerosis (4.3%) and found no *Encephalitozoon* spp. [19]. The detection of *E. bieneusi* in another immunocompromised population from the same geographical area provides additional regional context for investigating its occurrence and molecular characteristics.

Accurate detection of microsporidia can be challenging. Microscopic methods are relatively inexpensive and widely available but have limited sensitivity, particularly when spore numbers are low, and generally cannot provide reliable species identification [1, 2, 20]. PCR‐based assays can improve detection and permit species‐level identification, making them useful for investigating microsporidian infections in high‐risk populations [1, 2, 20, 21]. Nevertheless, molecular screening is not routinely available in many resource‐limited settings, and epidemiological information on circulating microsporidian species remains limited. For *E*. *bieneusi*, the internal transcribed spacer (ITS) region is the principal marker used for genotyping and investigating genetic diversity and host associations [12, 22–24], whereas the more conserved small subunit ribosomal RNA (SSU rRNA) gene is suitable for species detection and broader phylogenetic comparisons [2].

The present study extends our previous molecular investigation of intestinal protozoan infections in the same cohort of PLWH, in which *Cryptosporidium parvum* was detected and genetically characterized [25]. That study focused on *C*. *parvum*, whereas the present investigation examines microsporidian species using microscopic and molecular methods. We aimed to determine the occurrence of microsporidian infections among PLWH in Kurdistan Province, identify the infecting species, and examine the genetic relationship of the detected *E*. *bieneusi* sequence with previously reported sequences from human, animal, and environmental sources.

## 2. Materials and Methods

### 2.1. Study Design and Participants

This cross‐sectional study included 161 PLWH registered at the Kurdistan Provincial Health Center between May 2019 and November 2022. Adults with confirmed HIV infection were eligible for inclusion. The study population was the same cohort previously investigated for *C. parvum* infection [25]. The previous study focused on the molecular detection and subtype characterization of *C*. *parvum*; in contrast, the present study examined microsporidian infection using microscopic and molecular methods. Written informed consent was obtained from all participants. The study was approved by the Ethics Committee of Kurdistan University of Medical Sciences (IR.MUK.REC.1397.176). A STROBE checklist was included in the reports of cross‐sectional studies (File S1).

The sample size was based on the number of eligible HIV+/AIDS patients who attended the Behavioral Disease Counseling Center during the study period, agreed to participate, and provided a stool specimen. A total of 161 participants were therefore enrolled using convenience sampling, with one stool specimen collected from each participant. Because all eligible individuals who attended the center during the study period and provided informed consent were included, and no exclusion criteria beyond confirmed HIV infection were applied, no formal participant flow diagram was generated. The number of individuals who refused participation was not systematically recorded.

Although the overall study period was May 2019 to November 2022, stool specimens and demographic information were collected during the first year of the study. Microscopic parasitological examinations were performed at the time of sample collection. Molecular analyses were performed after the completion of sample collection and included all 161 stool specimens using the same DNA extraction and PCR protocols throughout the study.

### 2.2. Sample Collection and Demographic Information

Stool samples and demographic information, including sex, place of residence, animal contact, and drinking water source, were collected by a trained technician. Stool specimens were preserved in 10% formalin (for microscopic detection) or in 70% ethanol (for DNA extraction and PCR) [25].

Detailed clinical and immunological information, including CD4 count, HIV viral load, ART status, duration of HIV infection, gastrointestinal symptoms, AIDS/WHO stage, recent opportunistic infections, and antimicrobial or antiparasitic treatment, was not available to the research team. The only participant‐related information available was a limited set of demographic and epidemiological characteristics provided by the technician at the Behavioral Disease Control Center of Kurdistan University of Medical Sciences. There were no missing observations for any of the demographic or exposure variables examined in this study.

### 2.3. Microscopic Examination

Screening for microsporidian spores was performed using the modified trichrome blue staining method under light microscopy [20, 26, 27]. Stool specimens were first concentrated using the formalin–ethyl acetate sedimentation method, and smears were prepared from the sediment. All slides were examined using a 100x oil‐immersion objective (1000x total magnification). The entire surface of each slide was examined, and a specimen was considered positive if more than one typical microsporidian spore was observed. Three trained parasitologists independently examined the slides. Positive and negative control slides were included for quality control. Any uncertain findings were reviewed by all three examiners and resolved by consensus. Microsporidian spores were identified based on their characteristic morphology, staining characteristics, and approximate size.

### 2.4. DNA Extraction and Nested PCR

DNA was extracted from stool specimens using a commercial kit (YTA, FavorGen, Taiwan) according to the manufacturer′s instructions, with the following modifications. Nested PCR targeting the microsporidian SSU rRNA gene was performed based on the protocol described by Kumar et al. [28] and our recent report [19]. Briefly, 1 *μ*L of DNA template at a concentration about 50 ng/*μ*L was used for both the primary and secondary PCR assays. The primary PCR was performed in a final volume of 20 *μ*L, whereas the secondary PCR was performed in a final volume of 30 *μ*L. In both reactions, each primer was used at 1.5 *μ*L of a 10 pmol/*μ*L working concentration. The complete reaction compositions and thermal cycling conditions are provided in the revised manuscript.

The primary PCR amplified a 779‐bp fragment of the microsporidian SSU rRNA gene using the PMicF/PMicR primer pair. The secondary PCR was performed in separate reactions using species‐specific primer sets for *E. bieneusi* (EnbF/EnbR) and *Encephalitozoon* spp. (EncepF/EncepR), generating expected amplicons of 440 and 630 bp, respectively. For the positive control, a previously characterized positive sample deposited in GenBank under accession number KY823417 was used. This positive control sample had been obtained in a previous study. For the negative control, distilled water was subjected to the DNA extraction procedure and subsequently included in the PCR assay to monitor for potential contamination during DNA extraction and amplification.

PCR products were analyzed by agarose gel electrophoresis using safe stain and visualized under UV illumination with a Gel DOC imaging system. Molecular weight markers were included to confirm the expected amplicon sizes.

To minimize contamination, all procedures were performed under aseptic conditions using DNase/RNase‐free consumables, sterilized pipettes, and cleaned laboratory surfaces. Positive and negative controls were included in each PCR run. Pre‐ and postamplification procedures were performed in separate rooms with dedicated pipettes and reagents. DNA extraction from negative controls were included in each batch. All positive PCR products were repeated in an independent run to confirm the result.

### 2.5. Sequencing and Phylogenetic Analysis

The molecular analysis in this study was primarily designed for detection and confirmation rather than for molecular genotyping or comprehensive molecular epidemiological characterization. PCR amplification targeted the SSU rRNA gene of *E. bieneusi* was used for sequencinf. Unlike loci such as the ITS, which are commonly used for genotyping and assessing genetic diversity [12], the SSU rRNA target used in our study was selected primarily as a diagnostic/detection target [2]. Furthermore, because the SSU rRNA locus used in this study is not an appropriate marker for *E. bieneusi* genotyping, sequencing all PCR‐positive samples would not have provided the same type of genotype‐level epidemiological information that could be obtained using a suitable genotyping marker such as the ITS region.

The obtained sequence was edited using Chromas software https://technelysium.com.au/wp/chromas/ [29] and deposited in GenBank under accession number PQ056729. Reference sequences were selected based on their highest BLAST similarity, host and source diversity, and availability of SSU rRNA sequences from Iran and neighboring regions. Sequences were aligned using ClustalW and the Kimura 2‐parameter algorithm in MEGA12 software. The final alignment length was 400 bp. Gaps and missing data were treated by pairwise deletion. The best fitting nucleotide substitution model was selected using the Bayesian information criterion (BIC) in MEGA12 software [28]. Phylogenetic analysis was conducted using the maximum‐likelihood method with 1000 bootstrap replicates [28]. Nodes with bootstrap support < 70% were considered uncertain and were not interpreted biologically.

### 2.6. Statistical Analysis

Data were analyzed using SPSS software (Version 20). Associations between infection status and demographic variables were assessed using Fisher′s exact test. Because several variables contained zero cells among PCR‐positive participants, conventional maximum‐likelihood logistic regression was affected by complete separation and was therefore considered inappropriate. We consequently reanalyzed the associations using Firth′s penalized logistic regression, which provides bias‐reduced estimates in the presence of sparse data and separation. The corresponding profile penalized‐likelihood 95% confidence intervals are reported in the revised Table 1. Fisher′s exact test was used for categorical comparisons because of the small number of PCR‐positive cases and sparse contingency tables. Given that only 10 participants were PCR‐positive, a multivariable logistic regression model was not retained because of the very small number of outcome events and the resulting risk of overfitting and unstable estimates. The revised analysis focuses on the descriptive 2 × 2 tables, Fisher′s exact tests, and Firth‐penalized univariable logistic regression estimates. A two‐sided *p* value ≤ 0.05 was considered statistically significant. Because microscopy and PCR were performed on the same stool specimens, the two methods were compared using McNemar′s exact test. Percent agreement and Cohen′s kappa were calculated to assess concordance.

**Table 1:** Association between demographic characteristics and PCR‐confirmed *E. bieneusi* infection (*n* = 161).

Variable	Total *N* (%)	PCR‐negative *N* (%)	PCR‐positive *N* (%)	Firth‐adjusted OR (95% CI)	*p* value^a^
Sex					
Male	151 (93.78)	143 (94.7)	8 (5.3)	0.20 (0.04–1.03)	0.12
Female	10 (6.22)	8 (80.0)	2 (20.0)	Reference	
Drinking water					
Purified	151 (93.78)	141 (93.4)	10 (6.6)	1.56 (0.07–32.62)	1.00
Untreated	10 (6.22)	10 (100)	0 (0)	Reference	
Animal contact					
No	155 (96.27)	145 (93.5)	10 (6.5)	0.94 (0.04–22.33)	1.00
Yes	6 (3.73)	6 (100)	0 (0)	Reference	
Residence					
Urban	153 (95.03)	143 (93.5)	10 (6.5)	1.24 (0.06–27.33)	1.00
Rural	8 (4.97)	8 (100)	0 (0)	Reference	

*Note:* Firth′s penalized logistic regression was used to address complete separation arising from zero cells. The reported 95% confidence intervals are profile penalized‐likelihood confidence intervals. Fisher′s exact test was used for categorical comparisons because of the sparse data.

^a^Fisher′s exact test.

## 3. Results

### 3.1. Participant Characteristics

A total of 161 PLWH were included in the study. Most participants were male (151/161, 93.8%) and resided in urban areas (153/161, 95.0%). The majority reported using purified drinking water (151/161, 93.8%) and did not regularly have contact with animals (155/161, 96.3%) (Table 1). No missing data were observed for the demographic or exposure variables presented in Table 1; therefore, all 161 participants were included in the analyses of these variables.

### 3.2. Prevalence of Microsporidia

Nested PCR detected microsporidia in 10 of 161 participants (6.2%), whereas microscopy identified eight positive specimens (4.9%). All PCR‐positive samples were identified as *E. bieneusi*, and no *Encephalitozoon* spp. were detected. Because both microscopy and PCR were performed on the same 161 stool specimens, the two methods were compared using a paired analysis. The concordance between the two methods is shown in Table 2. Both methods were positive in seven specimens; microscopy was positive, but PCR was negative in one specimen; and PCR was positive, but microscopy was negative in three specimens. Both methods were negative in 150 specimens. The overall percent agreement was 97.5%, with a Cohen′s kappa of 0.765 (95% CI: 0.54–0.99), indicating good agreement. McNemar′s exact test showed no statistically significant difference in the positivity rates of the two methods (*p* = 0.625).

**Table 2:** Concordance between microscopy and nested PCR for the detection of microsporidia in 161 PLWH.

Samples	PCR‐positive	PCR‐negative	Total
Microscopy positive	7	1	8
Microscopy negative	3	150	153
Total	10	151	161

*Note:* Percent agreement: 97.5%; Cohen′s kappa: 0.765 (95% CI: 0.54–0.99); McNemar′s exact test: *p* = 0.625.

### 3.3. Factors Associated With Infection

No statistically significant associations were observed between *E. bieneusi* infection and the demographic characteristics examined, including sex, place of residence, animal contact, and drinking water source (Table 1). For drinking water source, animal contact, and residence, zero PCR‐positive cases in one category resulted in complete separation; therefore, the Firth‐adjusted odds ratios had very wide confidence intervals and should be interpreted with caution. The small number of PCR‐positive cases (*n* = 10), together with the highly unbalanced distribution of several exposure variables (animal contact: 6/161; rural residence: 8/161; untreated water: 10/161), limited the precision and interpretability of the association estimates, particularly for animal contact. These analyses are exploratory and severely underpowered, and the absence of statistically significant associations should not be interpreted as evidence of absence of association.

### 3.4. Molecular Characterization

Sequence analysis demonstrated 98%–100% nucleotide identity with *E. bieneusi* sequences available in GenBank. Phylogenetic analysis revealed that the current isolate clustered with previously reported sequences from human, animal, and environmental sources, indicating phylogenetic relatedness among these isolates. Several nodes in the tree had low bootstrap support, and the phylogenetic placement of the sequence from this study should therefore be interpreted with caution. However, this clustering alone does not provide sufficient evidence to establish zoonotic transmission (Figure 1).

**Figure 1:**
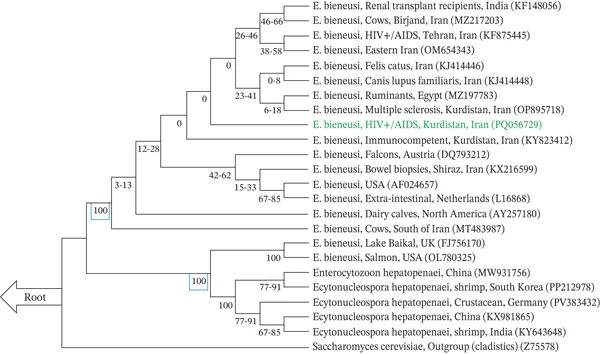
Phylogenetic relatedness between the human‐derived sequence and sequences reported from animal and environmental sources. Phylogenetic relationships of *E. bieneusi* sequences from this study and previously published sequences from GenBank. The tree was constructed using the maximum‐likelihood method with 1000 bootstrap replicates. The sequence from this study (PQ056729) is shown in bold and highlighted in green. Bootstrap values ≥ 70% are shown at the nodes. The evolutionary model used was Kimura 2‐parameter. The final alignment length was 400 bp. *Saccharomyces cerevisiae* (Z75578) was used as the outgroup. The sequence clustered with previously reported sequences from human, animal, and environmental sources. This phylogenetic relationship indicates genetic similarity but does not establish a zoonotic transmission pathway.

## 4. Discussion

This study provides molecular evidence of *E. bieneusi* infection among PLWH in Kurdistan Province, Northwest Iran. The molecular prevalence of 6.2% is consistent with the range previously reported among PLWH in Iran (6.0%–23%) [14–18], although it is lower than prevalences reported in some other regions, such as Turkey (17%) [29] and Ecuador (18.8%) [30]. Differences in prevalence between studies may reflect variations in patient characteristics, immune status, antiretroviral therapy coverage, diagnostic methods, geographical settings, and contact with animals.

PCR detected a higher number of positive specimens than microscopy, although the difference was not statistically significant in this paired comparison (McNemar′s exact test, *p* = 0.625). Nevertheless, the higher detection rate of PCR is consistent with previous studies demonstrating the superior sensitivity of molecular methods compared with conventional microscopy, particularly in patients with low parasite burdens [31, 32]. These findings support the incorporation of molecular techniques into the diagnostic evaluation of PLWH with suspected intestinal parasitic infections, especially when routine microscopy is negative but clinical suspicion remains high.

All PCR‐positive specimens were identified as *E. bieneusi*. This finding is consistent with the predominance of *E. bieneusi* among human intestinal microsporidian infections reported in different settings [1, 2, 4]. It is also consistent with the recent study from Kurdistan Province involving patients with multiple sclerosis, in which *E. bieneusi* was detected in 4.3% of participants and no *Encephalitozoon* spp. were detected [19]. The consistency between these two studies, conducted in different immunocompromised populations from the same region, supports the continued circulation of *E. bieneusi* in western Iran, although the studies cannot establish differences in prevalence between HIV and multiple sclerosis populations.

The predominance of *E. bieneusi* over *Encephalitozoon* spp. in stool‐based molecular surveys may partly reflect differences in their tissue tropism and patterns of infection. *E. bieneusi* is predominantly associated with intestinal infection, whereas *Encephalitozoon* spp. can involve a broader range of tissues and may cause disseminated disease, particularly in severely immunocompromised individuals [1, 2, 4]. These differences may influence the likelihood of detecting each group in fecal specimens, although the relative contribution of tissue tropism, immune status, and other clinical factors remains uncertain.

The sequence obtained in the present study showed high similarity to *E. bieneusi* sequences reported from human, animal, and environmental sources. This observation is relevant to the epidemiology of *E. bieneusi* in a region where livestock production and human–animal contact are common, although the limited number of participants without animal contact prevented a meaningful assessment of this exposure as a risk factor. However, this finding must be interpreted with caution. Only one PCR‐positive specimen was successfully sequenced, and the SSU rRNA marker is highly conserved and provides limited discriminatory power for *E*. *bieneusi*. High sequence similarity in this region indicates genetic relatedness but does not demonstrate zoonotic transmission or establish a specific epidemiological link. The phylogenetic findings are therefore compatible with, but do not demonstrate, shared transmission sources.

The lack of significant associations between infection and the demographic variables examined should be interpreted in light of the small number of positive cases. With only 10 PCR‐positive participants, the study had limited power to detect associations, particularly for exposure variables with little variation. The absence of an observed association with animal contact therefore does not exclude a role for animal exposure. A recent molecular study from the same region reported an association between animal contact and *E. bieneusi* infection among patients with multiple sclerosis, although the populations and study designs differed [19]. As reported elsewhere [12, 22], microsporidian infection in PLWH is likely influenced by multiple factors, including immune status, environmental exposure, sanitation, and access to effective antiretroviral therapy, rather than by demographic characteristics alone.

Although *E. bieneusi* predominantly causes intestinal infection [1, 22], extraintestinal involvement [33], including biliary [34], respiratory [35–37], and urinary tract infection [38], has been reported in severely immunocompromised individuals, particularly those with advanced HIV infection. The present study was based on stool specimens and therefore provides no information on extraintestinal infection.

The principal strength of this study is the use of complementary microscopic and molecular approaches in a defined cohort of PLWH from a region for which molecular data on microsporidia remain limited. The study also adds to our previous investigation of *C. parvum* in the same cohort [25] and to the recent evidence of *E. bieneusi* infection among patients with multiple sclerosis from the same geographical area [19]. These studies provide a broader regional context for intestinal microsporidian and protozoan infections in immunocompromised populations.

## 5. Limitations

Several limitations should be considered when interpreting the findings of this study. First, the cross‐sectional design and the use of a single stool specimen from each participant may have reduced the detection rate because spore shedding can vary over time. Second, participants were recruited through convenience sampling, and no formal a priori sample‐size calculation was performed. The small number of PCR‐positive cases (*n* = 10) limited the statistical power to assess potential risk factors and precluded multivariable regression. These analyses are exploratory and severely underpowered, and the absence of statistically significant associations should not be interpreted as evidence of absence of association. Furthermore, the highly unbalanced distribution of some exposure variables, particularly animal contact (reported by only six of 161 participants), limited the precision and interpretability of the corresponding association estimates. Findings related to animal contact should therefore be interpreted with caution.

Only one of the 10 PCR‐positive amplicons was successfully sequenced. Consequently, the genetic diversity of *E. bieneusi* in this population could not be adequately characterized, and the molecular epidemiological findings should be considered preliminary. In addition, the use of the SSU rRNA gene, rather than the ITS region commonly used for *E. bieneusi* genotyping, limited the genetic resolution of the analysis. No concurrent animal or environmental specimens were collected; therefore, the phylogenetic relationship observed in this study cannot establish or confirm a specific zoonotic transmission pathway.

Another important limitation was the absence of detailed HIV‐related clinical and immunological information, including CD4+ T‐cell counts, HIV viral load, antiretroviral therapy status, duration of HIV infection, diarrhea or other gastrointestinal symptoms, AIDS status or WHO clinical stage, and recent opportunistic infections. The unavailability of these data limited the assessment of clinical and immunological factors potentially associated with microsporidian infection. Finally, although the overall study period extended from May 2019 to November 2022, stool specimens and demographic information were collected during the first year of the study. Detailed information on antiretroviral therapy and temporal changes in HIV treatment practices was not available to the research team.

## 6. Conclusions

This study identifies *E. bieneusi* as the only microsporidian species detected among PLWH in Northwest Iran, with a molecular prevalence of 6.2%. Molecular screening detected a higher number of positive specimens than microscopy, although this difference was not statistically significant in the paired analysis (*p* = 0.625). However, because only a single SSU rRNA sequence was obtained, the molecular characterization remains preliminary. Further studies incorporating animal and environmental sampling and more discriminatory genetic markers, particularly the ITS region, are warranted to clarify the epidemiology and transmission of *E. bieneusi* in Northwest Iran.

**Nomenclature**
AIDS: acquired immunodeficiency syndrome
CI: confidence interval
HIV: human immunodeficiency virus
ITS: internal transcribed spacer
OR: odds ratio
PCR: polymerase chain reaction
PLWH: people living with HIV
SSU rRNA: small subunit ribosomal RNA

## Author Contributions

F.B.: conceptualization, methodology, investigation, and data analysis. E.S.: methodology and investigation. Y.M.: methodology and investigation. K.K.: data analysis. A.A.: conceptualization, data analysis, and manuscript preparation and editing. M.K.: conceptualization, validation, and project administration.

## Funding

No funding was received for this manuscript.

## Disclosure

All authors reviewed and approved the final manuscript.

## Ethics Statement

The study was conducted in accordance with the ethical principles of the Declaration of Helsinki and its subsequent amendments. This study was reviewed and approved by the Ethics Committee of Kurdistan University of Medical Sciences (IR.MUK.REC.1397.176).

## Consent

Written informed consent was obtained from all participants before enrollment in the study.

## Conflicts of Interest

The authors declare no conflicts of interest.

## Supporting information


**Supporting Information** Additional supporting information can be found online in the Supporting Information section. File S1: STROBE Statement—checklist of items that should be included in reports of *cross-sectional studies*.

## Data Availability

The anonymized dataset generated and analyzed during the current study is available upon reasonable request, subject to ethical restrictions imposed by the Ethics Committee of Kurdistan University of Medical Sciences.
